# Estimating the Potential Impacts of Large Mesopredators on Benthic Resources: Integrative Assessment of Spotted Eagle Ray Foraging Ecology in Bermuda

**DOI:** 10.1371/journal.pone.0040227

**Published:** 2012-07-03

**Authors:** Matthew J. Ajemian, Sean P. Powers, Thaddeus J. T. Murdoch

**Affiliations:** 1 Department of Marine Sciences, University of South Alabama, Mobile, Alabama, United States of America; 2 Dauphin Island Sea Lab, Dauphin Island, Alabama, United States of America; 3 Bermuda Reef Ecosystem and Mapping Programme, Bermuda Zoological Society, Flatts, Bermuda; National Institute of Water & Atmospheric Research, New Zealand

## Abstract

Declines of large sharks and subsequent release of elasmobranch mesopredators (smaller sharks and rays) may pose problems for marine fisheries management as some mesopredators consume exploitable shellfish species. The spotted eagle ray (*Aetobatus narinari*) is the most abundant inshore elasmobranch in subtropical Bermuda, but its predatory role remains unexamined despite suspected abundance increases and its hypothesized specialization for mollusks. We utilized a combination of acoustic telemetry, benthic invertebrate sampling, gut content analysis and manipulative experiments to assess the impact of spotted eagle rays on Bermudian shellfish resources. Residency and distribution of adult spotted eagle rays was monitored over two consecutive summers in Harrington Sound (HS), an enclosed inshore lagoon that has historically supported multiple recreational and commercial shellfish species. Telemetered rays exhibited variable fidelity (depending on sex) to HS, though generally selected regions that supported relatively high densities of potential mollusk prey. Gut content analysis from rays collected in HS revealed a diet of mainly bivalves and a few gastropods, with calico clam (*Macrocallista maculata*) representing the most important prey item. Manipulative field and mesocosm experiments with calico clams suggested that rays selected prey patches based on density, though there was no evidence of rays depleting clam patches to extirpation. Overall, spotted eagle rays had modest impacts on local shellfish populations at current population levels, suggesting a reduced role in transmitting cascading effects from apex predator loss. However, due to the strong degree of coupling between rays and multiple protected mollusks in HS, ecosystem-based management that accounts for ray predation should be adopted.

## Introduction

Declines in large sharks have been demonstrated in the northwest Atlantic for some time [1]. Evidence of shark declines also pervades subtropical oceanic islands in this region, where even low fishing pressure by either artisanal or subsistence fishing can have significant negative effects on populations of these slow-growing apex predators [2]. Reduction in top predators like sharks has been suggested to cascade through the ecosystem to basal resources such as shellfish. For example, recent meta-analyses demonstrated that cownose rays (*Rhinoptera bonasus*) transmit the effects of large shark declines down to bay scallop (*Argopecten irradians*) populations due to the specialist and density-dependent foraging behavior of the rays [3], [4]. However, the ecological role and potential impacts of many other large mesopredatory rays remain unknown due to the difficulty in studying these large and highly mobile animals.

The spotted eagle ray (*Aetobatus narinari*) is a large tropical to warm-temperate ray that consumes benthic invertebrates and is considered highly migratory [5]. Quantitative studies of the spotted eagle ray diet are limited, but suggest molluscivory throughout the species’ range when combined with anecdotal observations. Off the coast of North Carolina, *A. narinari* appears to specialize on clams [6], [7], whereas queen conch (*Strombus gigas*) may dominate the diet of individuals found in the Bahamas and Caribbean [8], [9]. In the Indopacific, *A. narinari* appears to be a key predator of giant clams (*Tridacna* and *Hippopus* spp.) and is considered a pest to farmers of these bivalves [10]. Despite their potentially wide-ranging impact to shellfish worldwide, published studies on the behavioral ecology of the spotted eagle ray consist of one tracking study and field observations of these animals off Bimini [11], [12], Bahamas and one quantitative description of the diet in the Indopacific [13]. However, no studies have examined foraging in this species relative to prey availability or density.

**Figure 1:**
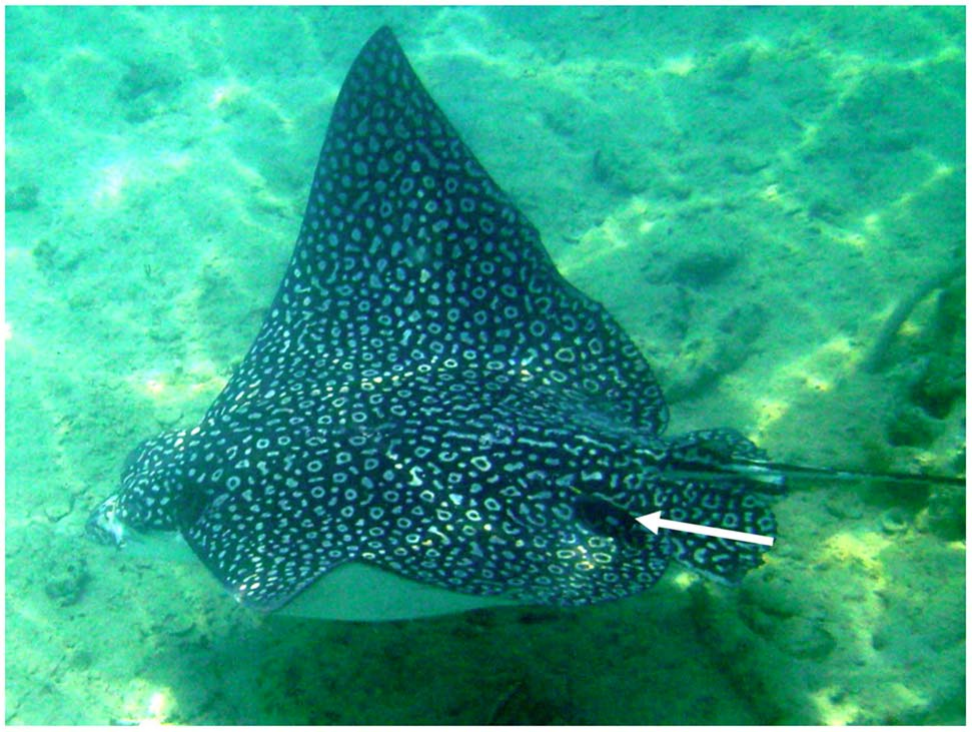
Photo of male spotted eagle ray *Aetobatus narinari* swimming with Lotek acoustic transmitter. Acoustic transmitters were externally secured to the dorsal “saddle” region, allowing for normal oscillatory swimming motion. White arrow points to transmitter location.

In subtropical Bermuda, the spotted eagle ray is reportedly common in seagrass beds and sand flats [14], but there are no data on its feeding habits, movements or habitat preferences in this region. Spotted eagle rays are prey to large sharks such as the great hammerhead *Sphyrna mokarran* [15], tiger *Galeocerdo cuvier* [16], and Caribbean reef *Carcharhinus perezi* (D.D. Chapman, pers comm.). Though fishery-independent data for sharks is lacking for the Bermuda Islands, landings suggest declining abundance of “dusky” sharks (*Carcharhinus galapagensis* and *C. perezi*) and increasing exploitation of larger tiger sharks (Bermuda Department of Fisheries, unpublished data). The potential “mesopredator release” of spotted eagle rays from large shark removals has raised concerns of apex predator declines cascading down to shellfish populations in Bermuda, many of which are protected (J.A Ward, Bermuda Conservation Services, pers. comm). Without knowledge of the habitat use and diet of these mesopredatory rays, however, the dynamics of potential cascading effects cannot be understood.

Despite its prevalence in the study of movements of other marine vertebrates, acoustic telemetry has only gained popularity in the study of batoids (skates and rays) over the last decade [12], [17], [18], [19], [20], [21]. Most of these acoustic telemetry studies of batoids have used manual tracking techniques to determine movement rates and use of physical habitat or monitoring to investigate general residency patterns. In spite of demonstrating the capability of tracking movements of these animals with acoustic telemetry, no studies have sufficiently linked movements or residency with prey abundance and/or distribution. Such questions can be addressed through integrating high-resolution acoustic technology with mapping of benthic communities.

In this study, we utilized a suite of approaches to examine the foraging ecology and habitat use of spotted eagle rays (*Aetobatus narinari*) in Harrington Sound (HS), Bermuda. Because of their highly mobile nature, we tracked and monitored rays with acoustic telemetry and analyzed their distribution relative to various benthic habitats in HS. During the monitoring period, benthic communities were sampled using bottom survey techniques to examine potential prey available and were compared to prey items consumed by rays. The spatial distribution and residency data of spotted eagle rays were then used to guide enclosure and field manipulation (exclosure) experiments aimed at quantifying the impact of rays on calico clam *Macrocallista maculata*, an abundant and protected bivalve species in HS.

**Figure 2:**
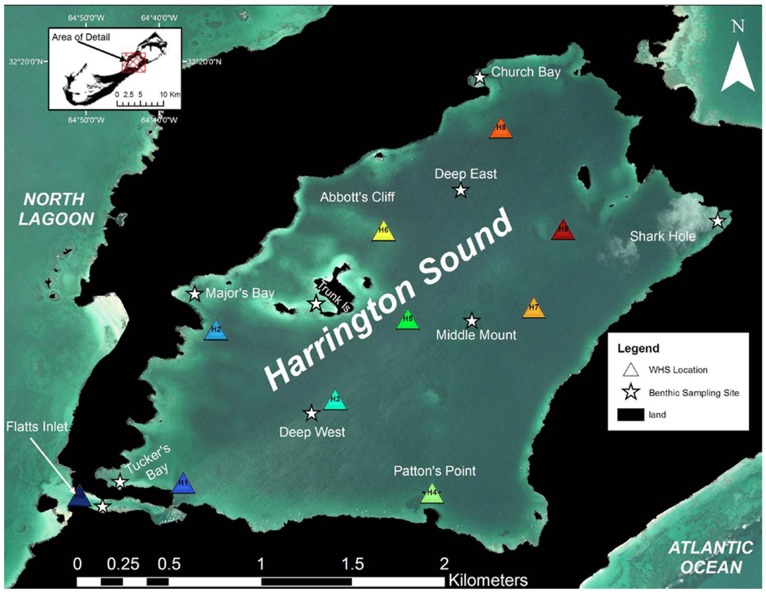
Map of Harrington Sound hydrophone layout and benthic sampling sites. Wireless Hydrophone Systems (WHS) are labeled from H0 (Flatts Inlet) - H9 and represented by colored triangles (2007 was H0–H1 only). White stars indicate locations of dive sites used for benthic sampling in 2008 and 2009 (note: benthic sampling dives were made at H4).

## Methods

### Ethics Statement

This study was conducted in accordance with the laws of the State of Alabama and under IACUC protocols (Permit # 05043-FSH) approved by the University of South Alabama. All efforts were made to minimize animal suffering during tagging procedures.

**Figure 3:**
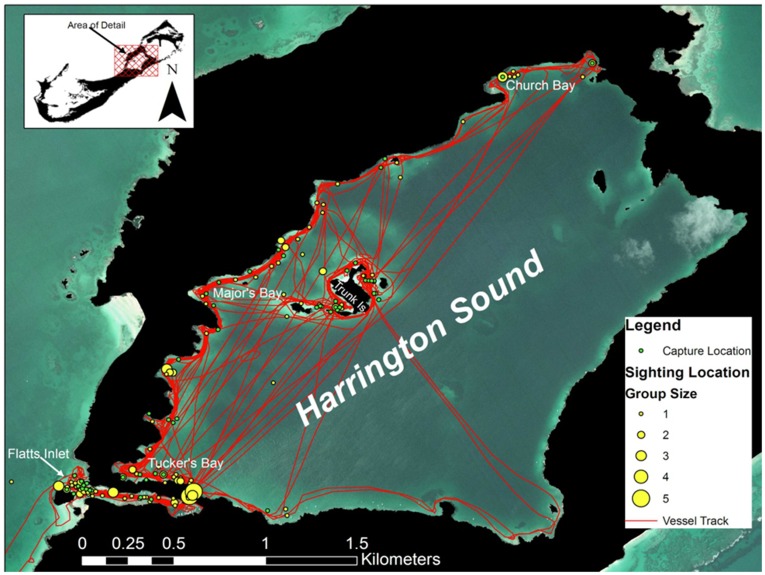
Sightings data for spotted eagle rays between Harrington Sound and Flatts Inlet (2007–10). Sightings (yellow circles) are scaled to ray group size (i.e. number of individuals) with green circles representing successful captures and survey “effort” denoted by the red vessel track.

### Study Area

The Bermuda Islands are limestone-capped volcanic pedestals situated within the Sargasso Sea and include several large inshore sounds and harbors. Harrington Sound (HS), the most land-locked of the inshore sounds, has a single navigable connection to the open ocean through Flatts Inlet (FI) (Thomas 2003). Surface temperatures of HS range from 16°C in winter to 30°C in summer. Mean tidal range in HS is 19 cm, lagging approximately 2 hr 53 min behind oceanic tides (Morris et al. 1977). HS is host to a wide variety of habitats, including seagrass beds (e.g. *Thalassia testudinum*) algal mats (*Cladophora prolifera*), sand flats, relic *Oculina* reefs, and rubble, mud and shell bottoms. A wide variety of infaunal and epifaunal benthic invertebrates characterize the heterogeneous benthos of HS, including commercially and recreationally important mollusks such as the calico clam, turkey wing (*Arca zebra*), Bermuda scallop *Pecten ziczac*, Atlantic Pearl Oyster (*Pinctada imbricada*) and conch (*Strombus costatus* and *S. gigas*). With the exception of turkey wing, all aforementioned mollusks have been protected in Bermuda waters since 1978 [22], though there have been no published attempts to estimate sources of natural mortality to these benthic invertebrates.

**Table 1:** Individual information from 18 acoustically tagged spotted eagle rays (2007–2010).

Release Date	Location	Sex	DW (cm)	MAP ID	Burst Interval (sec)	Warranty Life (d)	Last Detected	Days Monitored
5/16/2007	FI	M	130	40300	2	59	6/10/2007	25
5/16/2007	HS	F	161	40100	2	59	6/9/2007	24
5/21/2007	FI	F	121	40000	2	59	7/15/2007	55
5/21/2007	FI	M	123	40200	2	59	7/18/2007	59
5/22/2007	FI	F	145	90	2	57	7/9/2007	48
5/22/2007	FI	M	120	93	2	57	7/26/2007	66
5/22/2007	FI	M	137	94	2	57	7/15/2007	55
5/22/2007	FI	F	150	92	2	57	7/27/2007	67
6/25/2008	HS	F	149	54656	3	53	7/11/2008	13*
6/25/2008	HS	M	120	54708	3	53	8/30/2008	63
6/26/2008	FI	F	99	54604	3	53	n/a	0
6/26/2008	HS	M	124	54760	3	53	8/20/2008	53
6/26/2008	HS	F	102	54500	3	53	8/16/2008	49
6/27/2008	FI	M	129	54552	3	53	7/1/2008	3*
6/27/2008	HS	F	145	96	3	318	8/30/2008	63
6/27/2008	FI	M	133	98	3	318	8/16/2008	49
6/28/2008	HS	F	170	95	3	318	8/30/2008	63
6/28/2008	HS	F	156	97	3	318	8/31/2008	64

Footnote 1: For release location, FI = Flatts Inlet; HS  =  Harrington Sound. Five digit MAP ID codes represent MA-TP-16-252 transmitters (37 g in air). Two digit MAP ID codes represent MAP 16_3 s transmitters (33 g in air). Tags determined to be shed early are indicated with asterisk (*). Note: Days monitored are estimated from the start of the monitoring period until the last detection day, unless the tag was shed early).

**Table 2:** Results of two-way ANOVA on residency behavior of acoustically tagged spotted eagle rays in Harrington Sound.

Variable	Source	DF	Sum of squares	Mean squares	F	Pr > F
HS Mean Proportional Residency	Year	1	0.056	0.056	1.243	0.289
	Sex	1	0.160	0.160	3.541	0.087
	Year x Sex	1	0.026	0.026	0.568	0.467
HS Maximum Proportional Residency	Year	1	0.007	0.007	0.054	0.820
	Sex	1	0.681	0.681	5.563	**0.038**
	Year x Sex	1	0.157	0.157	1.284	0.281

Footnote 2: Items in bold represent significant *p*-value at 0.05.

**Figure 4:**
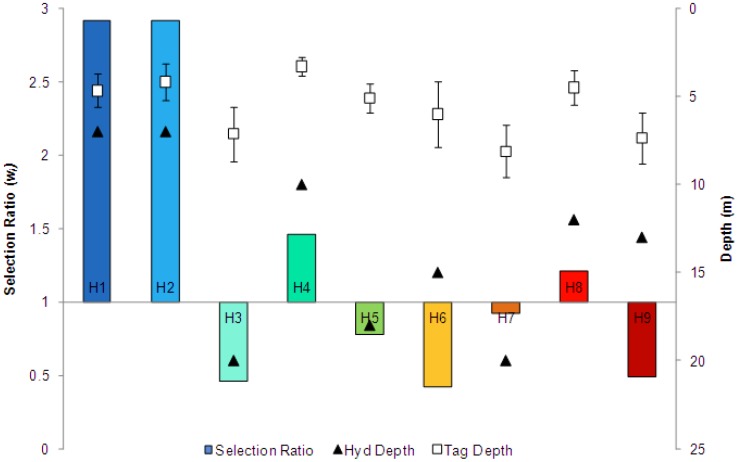
Vertical bar chart of tagged ray selection ratios for each Harrington Sound hydrophone station. Colored vertical bars represent selection ratios (left y-axis) at each hydrophone station, labeled near x-axis. Hydrophone depth is represented by black triangle (right y-axis). Mean tag depth is indicated by white squares (right y-axis) with standard error bars using individuals as replicates.

### Animal Capture

To better understand the foraging ecology of spotted eagle rays and their impacts on native shellfish populations, we collected 55 individual rays from the waters of Harrington Sound and Flatts Inlet between 2007 and 2010. Spotted eagle rays were visually located during daylight hours (0700–1900) from a moving skiff. Once located, a nylon seine net (100 m×5 m) was corralled around the animals and the encircled rays were brought aboard. Captured animals were measured (disk width, disk length, and weight), photographed for future identification, and assessed for level of sexual maturity when possible. Rays then received one of four treatments prior to release with an external tag: 1) fitted with an acoustic transmitter (n = 18), 2) pulsed gastric lavage (n = 18), 3) transported to enclosure pen for foraging experiments (n = 7), or 4) given no additional treatment (n = 12).

**Figure 5:**
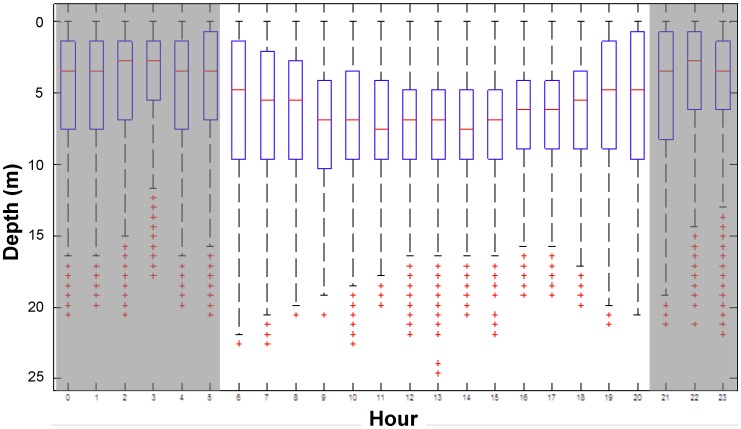
Hourly box and whisker plots of depth distribution for rays fitted with pressure sensor transmitters. Horizontal red lines represent mean depth, and blue boxes represent upper and lower quartiles. Whiskers represent maximum and minimum values, while red crosses represent outliers.

**Figure 6:**
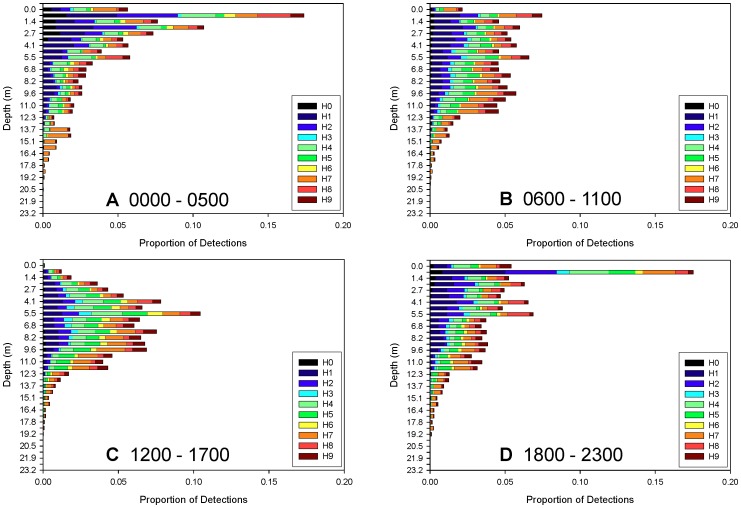
Stacked bar plots of vertical and horizontal habitat use for rays fitted with pressure sensor transmitters. Horizontal bars represent the proportion of detections at various depths of tagged animals (n = 6) within Harrington Sound during the 2008 monitoring period. Data are displayed in four 6-hour intervals: A –0000–0500, B –0600–1100; C –1200–1700; and D –1800–2300. Bars are color coded by hydrophone as in previous figures.

### Acoustic Telemetry

Acoustic telemetry was employed to determine habitat use and residency of spotted eagle rays in Harrington Sound. Acoustic transmitters were wrapped in a neoprene sleeve to reduce abrasion of the ray’s soft skin and attached to monofilament dart tags using cable ties and superglue. Following Silliman and Gruber (1999), tags were secured to the dorsal “saddle” region of spotted eagle rays (Figure 1). In each sampling year, two types of transmitters were used. In 2007, four animals were fitted with Lotek MA-TP16_252 transmitters (16×84 mm, 34 g in air) and four were fitted with MAP 16_3 s transmitters (16×82 mm, 33 g in air). The MA-TP16_252 transmitters transmitted an ID code, as well as sensor data for pressure (0–50 psi). The MAP 16_3 s transmitters only transmitted an ID code. Due to the short duration of the study in 2007, transmitters were prepared with a burst interval of 2 sec, translating to an estimated battery life between 53 and 59 days (MA-TP16_252 and MAP 16_3 s, respectively). In June of 2008, 10 individual rays were tagged and monitored from an acoustic array deployed across Harrington Sound. Six animals were fit with Lotek MA-PM-16_252 transmitters (16×84 mm, 34 g in air), which transmitted an ID code and sensor data for pressure (0–50 psi) and motion (0, 1) (Protocol S1). In addition, four individuals were tagged with MAP 16_3 s ID only transmitters coded to transmit every 3 sec (318 d battery life). All acoustic tags operated on a frequency of 76.8 kHz.

**Figure 7:**
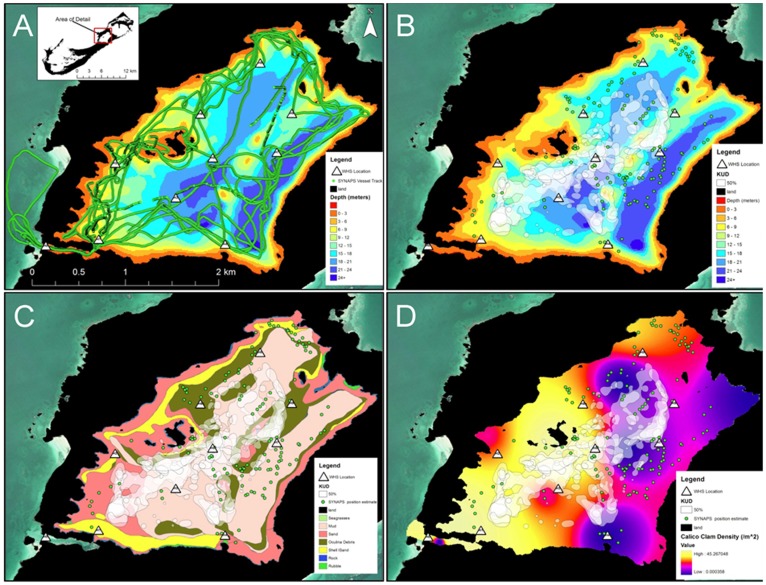
Maps of fine-scale habitat use of spotted eagle rays in Harrington Sound. Hydrophone detections from SYNAPS vessel tracks (A) and WHS 3050 power detection records were used to estimate tag position estimates (green circles) and centers of activity (50% KUD, white polygons), respectively, in B–D. Tag positions and COAs are overlaid onto bathymetry (B), bottom habitat (C) and interpolated calico clam density (D). Maps A-C are based on Thomas’ (2003) benthic survey of HS.

**Figure 8:**
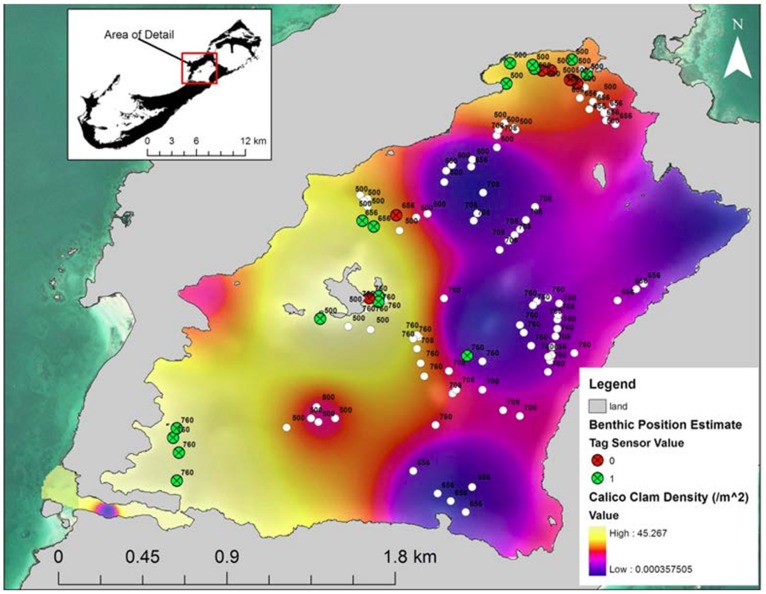
Map of potential benthic habitat use for pressure sensor tags in relation to prey density. SYNAPS position estimates for sensor tags 54500, 54656, 54708, and 54760 are overlaid onto interpolated calico clam densities. Motion values are color coded (0 = no motion, red; 1 = motion, green) and displayed for positions estimated within 1 m of the benthos. All other positions are displayed as white circles. The last 3 digits of each tag ID are displayed for each position.

**Table 3:** Track details from SYNAPS transects conducted in 2008.

TRACK INFO					TAG ID								
Track Session	Date	Track Start	Track End	Track Hrs	95	96	97	98	54500	54656	54708	54760	Total
1	7/2/2008	15∶02	17∶02	2∶00	1	3	0*	0*	2	1	3	0	10
2	7/3/2008	7∶58	10∶08	2∶10	2	1	1	4	11	7	0*	8	34
3	7/3/2008	12∶27	14∶29	2∶02	0*	2	2	1	13	4	3	8	33
4	7/3/2008	17∶33	19∶40	2∶07	1	2	2	2	3	4	1	4	19
5	7/4/2008	8∶44	10∶40	1∶56	0*	2	0*	2	1	3	1	8	17
6	7/4/2008	13∶05	15∶22	2∶17	2	0*	1	0*	1	0*	6	4	14
7	9/19/2008	13∶04	15∶05	2∶01	0	0*	0	0*	0*	0	0	0	0
8	9/20/2008	12∶15	14∶24	2∶09	0	0*	0*	0	0	0	0	0	0
9	9/21/2008	10∶40	12∶22	1∶42	0	0*	0*	0	0	0	0	0	0
				**18∶24**									**127**

Footnote 3: Tracking information is listed on the left, and the number of convergences for position estimates produced by SYNAPS is documented on right for each transmitter. Tag ID’s marked with an asterisk (*) represent sessions where the tag was detected, but there were insufficient detections to produce a convergent position estimate (CPE). Sum of track hours and CPEs are indicated in bold. Tag 54552 was removed from the table because it was considered a detached tag before the start of tracking sessions.

Acoustic monitoring array size, deployment length and locations differed between 2007 and 2008. In the 2007 study, 6 total Lotek Wireless Hydrophone Systems (WHS) 3050 (Lotek Inc.) were utilized; 5 throughout Harrington Sound (HS) and one unit at the junction between HS and Flatts Inlet. Hydrophones were straddled on either side of the junction at Flatts Inlet to serve as “gatekeepers”, which monitored the ingress and egress of the rays. The full 6 hydrophone array monitored tagged animal movements across HS from 21 May 2007 to 25 May 2007 with the two gatekeepers remaining until 28 July 2007 between FI and HS. All WHS 3050 units were vertically mounted to PVC poles secured to concrete moorings along the sea floor. In 2008, the acoustic array was expanded to 10 total WHS units, and included 9 WHS units in HS and one at the mouth of FI to serve as a gatekeeper (Figure 2). Monitoring within Harrington Sound occurred from 28 June 2008–31 Aug 2008.

**Table 4:** Benthic sampling site and abundance data for dominant infauna and epifauna from 2008.

Site	Depth (m)	Habitat Type	Infauna Species	Dominant Species	Max Density(individuals×m^−2^)	Epifaunal Species	Dominant Species	Site Density (individuals×m^−2^)
Tuckers Bay	2.7	Sand	5	*Macrocallista maculata*	45.8	3	*Pinctada imbricata*	25
Church Bay	3.0	Sand/cladophora	3	*Codakia orbicularis*	29.2	2	*Lytechinus variegatus*	12
Trunk Island	4.2	Sand	3	*Macrocallista maculata*	24.4	2	*Lytechinus variegatus*	3
Flatts Inlet	3.3	Sand	2	*Gouldia cerina*	12.5	0	N/A	N/A
Majors Bay	4.2	Sand	2	*Gouldia cerina*	16.7	3	*Pinctada imbricata*	20
Middle Mount	8.4	Shell	5	*Arca zebra*	12.5	3	*Lytechinus variegatus*	8
Patton’s Point	8.7	Sand/Shell	6	*C. orbicularis, A. zebra*	16.7	3	*Lytechinus variegatus*	20
Shark Hole	17.7	Silt/Mud	4	*Tellina laevigata*	12.5	0	N/A	N/A
Deep East	17.7	Silt/Mud	0	N/A	0	0	N/A	N/A
Deep West	20.1	Mud	2	*M. maculata, C. orbicularis*	4.2	0	N/A	N/A

**Table 5:** Benthic fauna collected from quadrat surveys across Harrington Sound and Flatts Inlet in 2008.

FaunaType	Class	Species	Count	Sites Observed	OverallDensity (/m^2^)	Habitat Density(/m^2^)	Habitat	Depth Range(m)
Infauna	BIVALVIA	***Macrocallista maculata***	15	4	6.9±14.9	15.6±20.2	sand/mud	2.7–20.1 (7.5)
		Codakia orbiculata*	12	3	5.6±10.4	16.7±12.5	sand/shell/cladophora	3.0–8.7 (5.0)
		*Gouldia cerina*	11	5	5.1±6.2	9.2±5.4	sand/shell/silt/mud	2.7–17.7 (7.3)
		*Arca zebra*	7	2	3.2±6.5	14.5±2.9	sand/shell	8.4–8.7 (8.6)
		*Tellina laevigata*	2	1	1.9±4.2	8.3±5.9	silt/mud/sand	2.7–17.7 (10.2)
		*Tellina listeria*	4	1	0.9±2.8	8.3±0.0	sand/shell	8.1
		Codakia orbicularis*	2	2	0.9±1.8	4.2±0.0	mud/silt	17.7–20.1 (18.9)
		***Anadara notabilis***	2	2	0.9±1.8	4.2±0.0	sand/shell	8.4–8.7 (8.6)
		*Macoma tenta*	1	1	0.5±1.4	4.2±0.0	sand	2.7
		***Tagelus divisus***	1	1	0.5±1.4	4.2±0.0	shell	8.4
	ECHINOIDEA	*Moira atropos*	4	2	1.9±4.2	8.3±5.9	sand/silt/mud	2.7–17.7 (10.2)
Epifauna	BIVALVIA	*Pinctada imbricata*	17	5	7.6±4.6	13.6±4.7	sand/shell/cladophora	2.7–8.7 (5.4)
	GASTROPODA	Strombus costatus*	7	3	3.1±3.2	9.3±1.5	sand/shell	2.7–8.4 (5.1)
	ECHINOIDEA	*Lytechinus variegatus*	10	3	4.4±4.2	3.3±1.5	shell/sand	2.7–8.7 (5.4)
	HOLOTHUROIDEA	*Actinopyga agassizi*	3	2	1.3±2.0	1.5±0.7	shell/sand	8.4–8.7 (8.6)
		*Isostichopus badionatus*	1	1	0.1±0.3	1.0±0.0	sand	2.7

Footnote 5: Habitat density refers to the average density at sites where each species was encountered, whereas overall density refers to the average density of each species across all sites. For each parameter, mean values plus one standard error are shown. Depth ranges are presented with average in parentheses. Species with asterisk * represent potentially consumed prey item (i.e. could not be identified to species) while those in bold were identified to species.

At the end of the monitoring period, data files (.txt format) were downloaded from the WHS 3050 units, filtered and imported into MATLAB (MathWorks, Inc.) for analysis and processing. To examine potential habitat preferences tagged rays within Harrington Sound, we performed a likelihood chi-square analysis on the observed number of detections recorded at each hydrophone with FishTel 1.4 (LabView, Inc.) telemetry analysis program. This program accounted for habitat availability (i.e. detection volume, Protocol S2) for each hydrophone, as well as the individual variability among rays (replicates) and determined a selection ratio (*w_i_*) for each hydrophone:
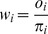
Where *o_i_*  =  proportion of the detections at hydrophone *i*, and π*_i_* = proportion of available resource units (i.e. volume) for that hydrophone. A *w_i_* value larger than 1 indicates a positive selection for the resource and a value less than 1 indicates avoidance [23]. To examine spatiotemporal behavior in vertical habitat use of tagged rays, we converted pressure sensor data to meters and binned detections at each depth by hydrophone over four time intervals. The time of day intervals were early AM (0000–0500 h), late AM (0600–1100 h), early PM (1200–1700 h) and late PM (1800–2300 h).

**Table 6:** Prey items observed in the gut content of spotted eagle rays from Harrington Sound.

Class	Family	Lowest Possible Taxon	%FO	%N
BIVALVIA	Veneridae	*Macrocallista maculata*	78.6	86.3
	Lucinidae	*Codakia* sp.	14.3	6.6
	Arcidae	*Anadara notabilis*	14.3	4.0
	Solecurtidae	*Tagelus divisus*	7.1	2.7
GASTROPODA	Naticidae	*Natica* sp.	7.1	0.3
	Strombidae	*Strombus costatus*	7.1	0.1

Footnote 6: Percent frequency of occurrence (% FO) and percent by number (% N) for various prey items consumed by spotted eagle rays. Prey items were identified to the lowest possible taxon (genus or species).

We explored the effect of tagging year (2007, 2008), sex and the interaction (year x sex) on Harrington Sound proportional habitat use (proportion of the monitoring period rays were detected in Harrington Sound; Protocol S3) and maximum residency interval (maximum time in Harrington Sound) of spotted eagle rays using univariate two-way ANOVA. We also examined the influence of ray size (i.e., disk width) on proportional residency in Harrington Sound with linear regression. Proportional data were arc-sine transformed prior to analysis, and ANOVA post-hoc analyses were performed using Tukey’s HSD [24]. All ANOVAs were performed using XLSTAT 10.0 (Addinsoft, Inc.).

**Figure 9:**
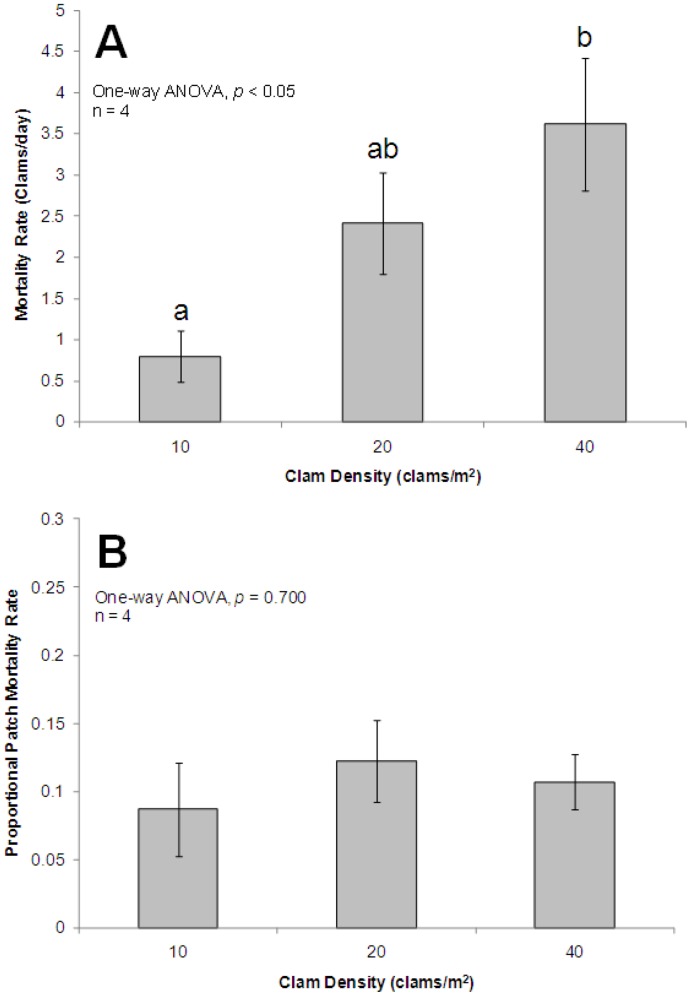
Bar charts of mortality among three clam tray densities from enclosure experiments. A  =  Mean ±1 SE of clam mortality rate (i.e. number of clams missing/trial days) across three densities (groups exhibiting significant differences are denoted by lowercase letters), B  =  Mean ±1 SE for proportional patch mortality rate (proportion of patch missing/day).

**Table 7:** One-way ANOVA table data from enclosure experiments.

Variable	Source	DF	Sum of squares	Mean squares	F	Pr > F
Clams eaten per trial	Model	2	137.167	68.583	5.523	**0.027**
	Error	9	111.750	12.417		
	Corrected Total	11	248.917			
Proportional mortality rate	Model	2	0.002	0.001	0.371	0.700
	Error	9	0.030	0.003		
	Corrected Total	11	0.032			

Footnote 7: Results are shown for effect of clam density on clam mortality rate and proportional patch mortality rate (proportion of clams killed per trial). Items in bold represent significant values at *p*<0.05.

Fifteen minute centers of activity (COA) were determined for acoustically tagged rays to examine use of the interior portion of the Harrington Sound monitoring array. Individual COA positions were determined using a weighted activity cell method developed by Yergey et al. (2012), which was adapted from Simpfendorfer et al. (2002):
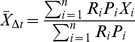


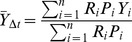
Where *R_i_* is the number of detections a hydrophone *i*, *P_i_* is the power of detections at hydrophone *i*, and *X_i_* and *Y_i_* are the *x* and *y* coordinates of hydrophone *i* [25], [26]. The COAs were only estimated for intervals in which individual tags were detected by three or more hydrophones. The kernel density estimator in Hawth’s Tools (http://www.spatialecology.com) was then used to examine overall distribution of COAs relative to various benthic habitats within the convex polygon of hydrophones in HS.

**Figure 10:**
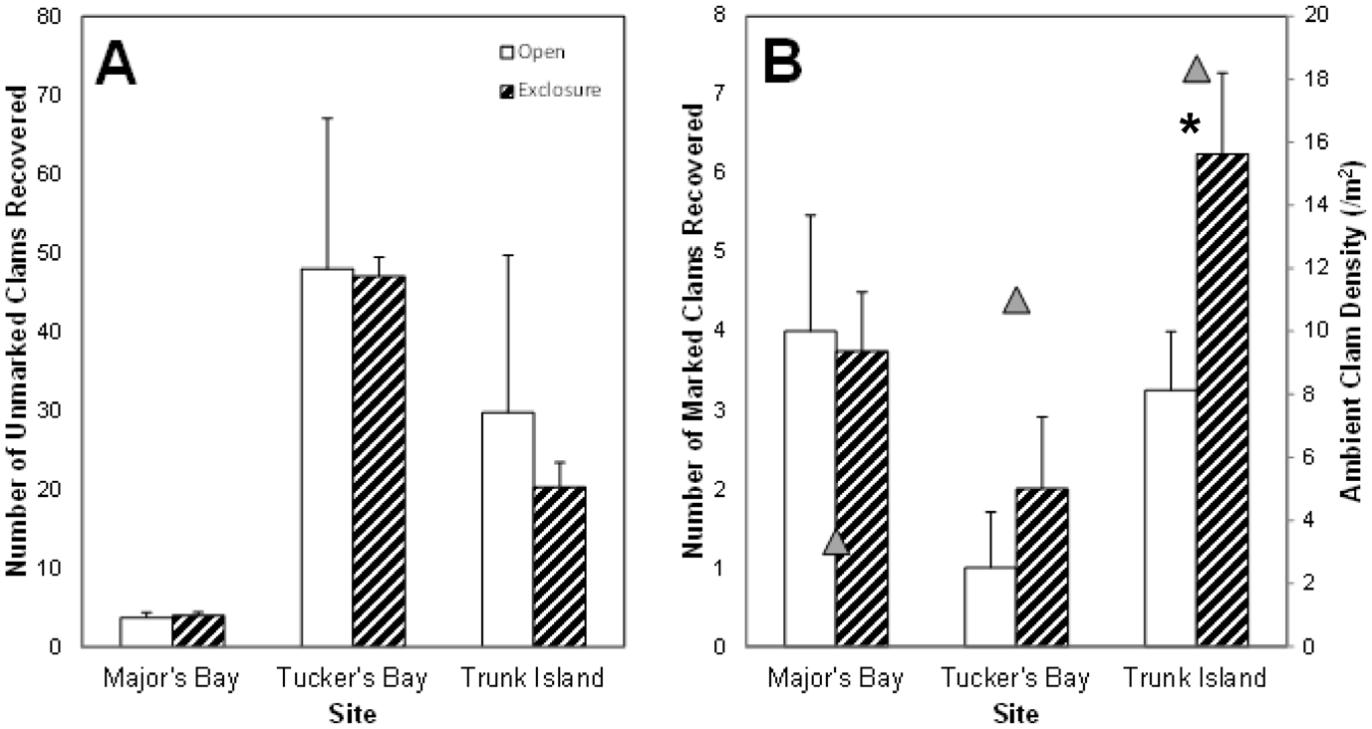
Vertical bar charts of calico clam recovery from field manipulation experiments. Unmarked (A) and marked (B) calico clam recovery comparisons between open (white) and exclosure (black and white diagonal strips) patches at three sites within Harrington Sound. In B, Ambient calico clam density is plotted for each site (gray triangles) along the secondary y-axis. * - denotes clam recovery was significantly different between patches at *p*<0.05 from t-test.

**Table 8:** Population estimation table of spotted eagle rays from Harrington Sound.

Date	#Captures(*M_t_*)	#Recaps(*R_t_*)	Free Marks(*F_t_*)	M_t_*F_t_ ^2^	R_t_*F_t_
May-07	8	0	0	0	0
Jul-07	3	0	8	192	0
Jun-08	10	1	11	1210	11
Sep-08	3	0	21	1323	0
Jun-09	8	0	24	4608	0
Oct-09	13	1	32	13312	32
Jul-10	10	2	45	20250	90
Totals	55	4	141	40895	133
				**N-hat**	**307**

Footnote 8: *M*_t_  =  the number of captured individuals at time *t*; *F*_t_  =  the number of free marks, or total individuals previously marked in the population at time *t*; *R*_t_  =  the number of recaptured individuals at time *t*; and N-hat  =  the estimated population size.

To estimate fine-scale (±10 m) habitat use by spotted eagle rays, we conducted a series of mobile tracking surveys (Protocol S4). Tracking surveys were conducted July 2–4, and in September 19–21, 2008. After data collection, Synthetic Aperture Positioning System (SYNAPS, Lotek Wireless, Inc.) software was used to estimate positions of the transmitting tags. Convergent position estimates were plotted in ArcMap 9.2, and localizations within various habitat types were extracted from digitized maps of Harrington Sound using Hawth’s Tools. To examine benthic habitat use of individuals tagged with depth-sensing transmitters, we used the intersect function in Hawth’s Tools to extract the position estimates within 1 m of the benthos.

### Benthic Sampling

In order to examine the distribution of prey available to spotted eagle rays, we conducted benthic quadrat surveys concurrently with acoustic monitoring at ten sites throughout Harrington Sound and Flatts Inlet (Figure 2). At each site, we haphazardly lowered a 50 cm×50 cm (0.25 m^2^) weighted PVC frame four times along a 25 m transect. At each quadrat we excavated the upper 15 cm of the area by hand and collected all epi- and infaunal invertebrates by passing excavated sediment through a 2 mm mesh bag. Excavated fauna were identified to species level and enumerated. For each species, we determined density per square meter by pooling the counts of the four 0.25 m^2^ quadrats per site. Due to the restricted spatial coverage of the benthic sampling, we interpolated densities of dominant benthic fauna using the inverse distance weighted (IDW) method in the Spatial Analyst extension of ArcMap 9.2. We elected IDW interpolation due to its utility in analyzing relatively sparse or irregularly spaced data sets (ESRI, Inc.). Interpolations were used to assess spatial trends in faunal abundance across HS and qualitatively compared with spotted eagle ray distribution.

### Diet Analysis

Gut contents of spotted eagle rays were sampled opportunistically using pulsed gastric lavage (PGL) on live animals. During PGL, a plastic tube was placed into the esophagus of the ray and a gentle stream of ambient water was run into the stomach to flush out the contents. This technique was a non-lethal alternative to sacrificing rays for dietary information. We verified the efficacy of this technique by sacrificing three individuals after PGL was conducted, and found that PGL successfully removed 100% of the gut content. All animals sampled survived the PGL procedure. Recovered gut content was identified from photos of local benthic invertebrate tissue. For each prey item, we calculated percent frequency of occurrence: 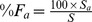 where *S*_a_ is the number of stomachs containing food group *a*, and S is the total number of stomachs containing food [28]. Using identifiable parts of mollusk tissue (e.g., muscular foot of clam), we also estimated the number of individuals for each prey item consumed (% *N*_a_).

### Enclosure Experiment

To examine small-scale foraging preferences of spotted eagle rays among heterogeneous prey densities, we conducted a series of patch selection experiments within an experimental enclosure. The enclosure measured 46 m×22 m and was 1–2 m in depth. The border of the enclosure was constructed from plastic fencing material with a 2.54 cm diameter styrofoam float line at the top and a lead-line sewn into the bottom. Floats were placed evenly at 5 m intervals across the top of the net to ensure flotation during high tide. Additional anchors were used to secure the enclosure in place. The substrate within the enclosure was primarily characterized by coarse sand with a few 10 m^2^ regions of limestone rock. Prior to experimentation, the sandy area within the enclosure was excavated by SCUBA divers to remove any ambient infauna and epifauna to a depth of 15 cm. Calico clams collected from nearby locations in Harrington Sound were distributed among 9 separate 1 m^2^ vexar screen trays filled with sand. During each trial, trays were randomly assigned a number between 1 and 3, and given a density of 10, 20 or 40 calico clams per square meter (these densities were chosen to reflect natural densities of these clams in nearby Harrington Sound). Calico clams were introduced to the surface of the trays 24 h after rays were placed inside the enclosure. Trays were checked daily for evidence of predation and restocked when necessary. Analyses of variance (ANOVA) were used to test the impact of clam density on the number of clams consumed and mortality rate (proportion of clams consumed per day). To better meet the assumptions of ANOVA, all proportional tray mortality rates were arc-sine square root transformed prior to analysis and checked for normality and heterogeneity of variances.

### Field Manipulation Experiment

To estimate the contribution of spotted eagle ray predation to calico clam mortality in Harrington Sound, we conducted a 3-month field study in 2009 where we introduced marked calico clams to unprotected (open) patches and protected (exclosure) patches. Four replicate open and exclosure patches (i.e., 8 patches) were constructed at 3 different sites (24 patches total): Major’s Bay, Tuckers Bay and Trunk Island (Figure 2). The three locations were chosen to reflect different levels of ambient calico clam density and because rays had been previously observed at these locations (Figure 3). Exclosures were 2 m×2 m (4 m^2^) square patches with 75 cm tall steel poles spaced 25 cm apart around the perimeter. These dimensions were chosen because all rays sampled were 60–170 cm in disk width, and thus were assumed to be capable of accessing open patches but too large to enter stockades. Yellow flagging tape was stretched across the top of the exclosure to deter animals from entering from above. Excavated “open” patches (4/site) were placed haphazardly nearby (≥3 m) the exclosures. Corners of the open patches were marked with short metal stakes and flagging tape. In July 2009, SCUBA divers excavated these patches of infauna and epifauna to a depth of 15 cm. Ten marked calico clams were placed haphazardly throughout patch and tethered to the substrate at least 50 cm from the edge. Patches were re-excavated in October 2009 using the same method and the number of marked clams and unmarked clams was recorded at each patch. Due to the high variability in clam mortality rates among sites, separate independent samples *t*-tests were run at each site comparing the number of marked-tethered clams recovered between exclosure and open patches. It was assumed that marked-tethered clams missing from patches were killed by predators. Shells that were emptied (i.e., octopus predation), but not crushed, were not considered killed by rays or smaller predators (crabs). Shells broken into large pieces were considered killed by crabs. The various species-specific fracture morphologies of calico clam shells were confirmed through tests with captive predators at the Bermuda Aquarium Museum and Zoo.

### Impact on Calico Clam Population

We used tag-recapture data to develop an approximate population estimate for spotted eagle rays in Harrington Sound. Population size was assessed with the Schnabel method, which is an extension of the single sample Lincoln-Peterson model of population estimation to include a series of samples 29]. Each sampling trip (2007–2010) was used as a “sample” because we captured, examined for previous marks, marked and released animals on each of these trips and revisited the same sites continuously throughout Harrington Sound and Flatts Inlet. We then used the following formula to estimate the population:
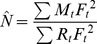
Where *M*_t_  =  the number of captured individuals at time *t*; *F*_t_ = the number of free marks, or total individuals previously marked in the population at time *t*; *R*_t_  =  the number of recaptured individuals at time *t*; and N-hat  =  the estimated population size. We used a modification of this index to incorporate movement rates of spotted eagle rays in a “closed” population model (Protocol S5).

Because daily rations of elasmobranchs (including batoid rays) range from 0.3–4.3% of body weight per day [30] and do not appear to exceed 3.0% for the related cownose ray [31], we assumed a 3% daily ration for related spotted eagle rays. This daily ration estimate, combined with an estimate of overall ray population size (population x mean residency in HS) and densities of primary prey across available habitat of Harrington Sound (determined from benthic sampling) was used to quantify summertime calico clam removal by spotted eagle rays.

## Results

### Catch Distribution

Spotted eagle rays were sighted along shallow sand flats <5 m in depth within Harrington Sound and Flatts Inlet (Figure 3). The majority of sightings (80%) were comprised of solitary individuals, though as many as five individuals were observed shoaling at a time. These larger groups were most evident at the connection between Flatts Inlet and Harrington Sound near Flatts Bridge. Several pairs and trios were also observed at the mouth of Flatts Inlet where the inlet connected with the North Lagoon. Successful captures were distributed throughout the northwest shoreline of Harrington Sound and included regions such as Church Bay, Major’s Bay, Trunk Island, and Tucker’s Bay. Several captures were also made towards the mouth of Flatts Inlet.

### Acoustic Telemetry

We monitored movements of four female (121–161 cm DW) and four male (120–137 cm DW) spotted eagle rays in 2007 between Harrington Sound and Flatts Inlet with gatekeepers only. In 2008, four males (120–133 cm DW) and six females (99–161 cm DW) were monitored across the extended Harrington Sound acoustic array. Monitoring periods ranged from 3–67 days; with 94% of tagged individuals detected by the acoustic array (Table 1). Neither sex nor year tagged was found to influence proportional habitat use (Table 2; *p*>0.05). However, when maximum residency was utilized as the response variable, a significant sex effect was found (Two-way ANOVA; *F*_1,14_ = 5.56, *p* = 0.038). Mean maximum residency (*m* = 37.9±6.6 d) for females was found to be significantly higher than mean maximum residency (*m* = 16.7±6.3 d) for males (*t-*test, *p*<0.05). No significant relationship was found between ray size and proportional residency in Harrington Sound (linear regression, r^2^ = 0.0001, *t* = 0.041, d.f. = 14, *p* = 0.968).

Based on 2008 acoustic monitoring data, spotted eagle rays habitat use was not evenly distributed throughout Harrington Sound (χ^2^ = 1251886.01, df = 64; *p*<0.0001). The highest number of proportional detections (detections per available volume) was recorded in the shallow southwest portion of the Sound. In this region, selection ratios were all greater than 1 (*w_H1_* = 2.921, *w_H2_* = 2.922, *w_H4_* = 1.461), indicating observed detections were greater than expected by the total available habitat (Figure 4). Relatively high use was also observed at H8 (*w_H8_* = 1.21) at the northeast corner of HS, which was also situated in shallow waters. Low use and thus avoidance (*w_i_* <1) was generally observed for deeper hydrophones of the interior of HS, and the lowest amount of total detections was recorded at the hydrophone stationed at Flatts Inlet (H0).

The rays fitted with pressure sensor tags were detected at all available depths within Harrington Sound (0–25 m). However, 90–95% of the acoustic detections occurred in the upper 10 m (Figure 5). Tag depth varied greatly with time of day; the highest proportion of detections occurred at or near the surface during the 0000–0500 h and 1800–2300 h periods. During these dark periods, the greatest amount of detections occurred at 0.7 m (Figure 6) suggesting rays were most often distributed in subsurface waters during low light conditions. A dramatic diel shift in ray vertical distribution occurred from surface to deeper waters during daylight hours. While vertical habitat use of rays appeared more evenly distributed during the late morning (0600–1100 h, Figure 6B), proportional use of the upper depths decreased dramatically in the afternoon (1200–1700 h) (Figure 6C). During the afternoon period, the vertical distribution of rays followed a bell curve with peak use at 5.5 m depth (Figure 6C). The density of ray detections was consistently high across the southwest portion of HS all hours of the day (H1, H2), though there was notable shift in movement towards the interior of HS during midday.

Combined COAs for all tagged rays showed high concentrations of use (i.e. overlapping 50% KUD) within sub-arrays H1-H2-H3 and H2-H3-H5, and between H7 and H9 (Figure 7B–D). Notably lower COA concentrations were observed within sub-arrays H6-H8-H9 and H1-H3-H4. Benthic habitats underlying the core use regions included mud, sand, *Oculina* debris and shell sand, all typically >15 m in depth. Primary prey density was generally high within the 50% KUD for most individuals, although the core use region between H7–H9 was devoid of clams (Figure 7D).

Mobile tracking transects using SYNAPS detected all tags recorded on the WHS array (Table 3) and effectively positioned tags both within and outside the confines of the minimum convex polygon of HS hydrophones (Figure 7A,B). The likelihood chi-square test on convergent position estimates found that the underlying habitats were not evenly utilized (χ^2^ = 84.83, df = 45; *p*<0.001). Similar to the qualitative COA analysis, towed hydrophone telemetry processed with SYNAPS showed rays used overlying waters of mud, relic *Oculina* debris, shell-sand and sand habitats, while fringing rock and rubble habitats of the immediate coastline were avoided (Figure 7C). However, error bars from 95% confidence intervals of selection indices overlapped for all habitats where rays were positioned, suggesting that there was no preference among the substrates. SYNAPS analysis of all tags positioned animals over a range of calico clam densities (Figure 7D). Subsequent analysis of depth sensor tag data showed that three (54500, 54656, 54760) of four individuals positioned by SYNAPS interacted with the substrate in regions characterized by high primary prey (calico clam) densities (Figure 8). Moreover, position estimates made over these shallow habitats included instances of reduced movement (i.e. sensor value = 0) for all three individuals. Use of the interior of the sound (i.e. surrounding region of seamounts) appeared consistent across several individuals during the tracking transects. However, individuals positioned in these regions generally occupied mid-depth or surface waters, with little evidence of substrate use (Figure 8).

### Benthic Sampling

Harrington Sound infaunal and epifaunal abundance and diversity was negatively correlated with water depth. Infaunal abundance was highest in sand or shell habitats with lower amounts collected in mud and silt (Table 4). Epifaunal species were not observed below 8.7 m and were similarly found at higher abundances over sand and shell habitat (Table 4). Across all sampling sites in Harrington Sound, calico clam *Macrocallista maculata* was the most abundant infaunal bivalve collected and achieved the highest maximum density per site (Tables 4, 5). Although encountered at lower average densities, the waxy gould clam *Gouldia cerina* was collected across the greatest number of sites of all benthic invertebrates and thus had the greatest average density (Table 5). Epifaunal species included the dense and pervasive Atlantic pearl oyster (*Pinctada imbricata*), milk conch (*Strombus costatus*), purple urchin (*Lytechinus variegatus*) and two species of sea cucumbers (Table 5).

### Diet Analysis

Opportunistic sampling of spotted eagle ray gut content revealed a diet dominated by mollusks (Table 6). Though sample size was low (n = 4), no food items were found in the gut content of individuals lavaged in Flatts Inlet. Contrastingly, all individuals collected in Harrington Sound (n = 14) possessed identifiable items in the gut, all of which were observed on benthic surveys in HS (Table 5). Total ingested material ranged between 0.4–8.5% of ray body weight (mean = 4.7±2.7%) for 9 individuals. Calico clam (*M. maculata*) was determined to be the prey item of highest importance, both by frequency (78.6%) and by number (86.3%) for 14 individuals (Table 6). In fact, five different individuals (both male and females) were collected with >100 calico clams in their gut content (max = 161 clams). Other notable but less important bivalves recorded in the diet included lucines (*Codakia* sp. –14.3% FO, 6.6%N), eared ark (*Anadara notabilis –*14.3% FO, 4.0% N) and purplish tagelus (*Tagelus divisus* –7.1% FO, 2.7% N). One individual ray (170 cm DW female) was found to consume two species of gastropods, the moon snail (*Natica* sp. –7.1% FO, 0.3% N) and milk conch (*Strombus costatus* 7.1%N, 0.1% N). Most individuals (80%) possessed a single prey item type in their gut content, with generally no shell fragments.

### Enclosure Experiment

Spotted eagle rays were initially offered calico clams and milk conch, but due to the lack of milk conch consumption by captive animals, experiments proceeded with calico clam only treatments. A significant clam density effect was found for total clams eaten (*F*_2,11_ = 5.523, *p*<0.05; Table 7). Bonferroni pairwise comparisons revealed significantly more clams were consumed in the high density treatment (mean = 10.5 clams/trial) than the lowest density (2.5 clams/trial; *p*<0.01) treatment, though medium density mortality (7 clams/trial) was not found to be different from the high or low density clam mortality rates (*p*>0.05; Figure 9A). When utilizing the same data set to examine the effect of calico clam density on proportional patch mortality rate, the ANOVA found no significant effect (*F*_2,11_ = 0.371, *p* = 0.700; Table 7, Figure 9B).

### Field Manipulation Experiments

After complete excavation in July 2009, unmarked calico clams colonized open and exclosure patches at the three manipulation sites by October 2009. Clam immigration (denoted by the number of unmarked clams) was highest at Tuckers Bay (Figure 10A) followed by Trunk Island, and Major’s Bay. Another trend was the substantially higher variability in the number of unmarked clams in the open patches at both Trunk Island and Tuckers Bay when compared to exclosures at these same sites. Despite this higher variability, patch type did not influence the recovery of unmarked clams at any of the three experimental sites in Harrington Sound (two-sample *t*-tests, *p*>0.05).

The recovery of marked-tethered calico clams was significantly influenced by patch type (exclosure vs. open) at the Trunk Island site (*t*(4) = 2.353; *p* = 0.019; Figure 10B) with higher recovery in exclosure stockade patches (6.75±0.75 clams/patch) than open patches (3.75±1.0 clams/patch). Ambient clam densities were also observed to be highest at Trunk Island compared to other sites in the 2009 benthic sampling period (mean = 18.3±3.5 clams · m^−2^). No significant patch effects were found at the Tuckers Bay (*t*(4)  =  −0.866; *p* = 0.386) or Majors Bay (*t*(4)  =  −0.662; *p* = 0.508) manipulation sites (Figure 10B), though chipped and open shells from smaller predators were noted.

### Calico Clam Impact

The calico clam population in Harrington Sound was estimated at 2.01±0.72×10^7^ individuals, given a total habitat area (sandy bottom <10 m) of 1.38 km^2^ and average density of 16.55± clams · m^−2^ across all habitats where clams were collected in 2008 and 2009. Using the average weight of 35 kg for all rays collected in HS, rays would consume 1.05 kg (3.0% of BW) of foodstuff per individual per day. For rays feeding on calico clams (mean clam tissue wet weight of 7.765 g from benthic sampling), this translated to a feeding rate of 135 clams per day. Of the 55 individual rays collected in Harrington Sound, four were identified as recaptures (7.3% recapture rate). All recaptures were large mature females (generally >150 cm disk width) originally tagged within Harrington Sound. Based on these recapture rates, the Schnabel method estimated 307 individuals inhabited Harrington Sound (Table 8). Given a 73.3% average residency level from acoustic monitoring, the eagle ray population would inhabit HS for 66 d period throughout the summer, during which they would consume 2.75×10^6^ clams (13.7% of the standing stock).

## Discussion

Our combined acoustic monitoring and tracking work showed that mobile spotted eagle rays did not evenly partition available habitat within a semi-enclosed lagoon, and exhibited a strong affinity to shallow (<10 m) sand and sand-shell bottom habitats. This core use area was situated among the highest densities of their preferred prey (calico clam) recorded in benthic surveys of Harrington Sound. Although our active tracking transects positioned spotted eagle rays over various other habitat types (mud, relic *Oculina*, etc.), they were rarely positioned at or near the benthos in these regions. The lack of substrate use in these habitats suggests they were not used for foraging purposes since eagle rays prey solely on benthic organisms. Along sandy habitats of Tuckers Bay, Trunk Island and Church Bay, on the other hand, tracked rays were observed on or near bottom. While these areas were indeed shallower, rays were never detected in the upper depth layer when positioned in these sandy regions. Conversely, rays were commonly positioned in surface waters overlying deeper regions of HS. Reduced movement of rays in the sand habitat, as indicated by motion sensor tags, may be indicative of foraging behavior as rays must cease forward swimming to excavate prey from the benthos.

Spotted eagle ray preference for sandy and shallow habitats was likely due to the greater prey availability; though benthic sampling revealed potential prey down to 20 m, the majority of infauna and epifauna were collected along a 5 m interval between 2.7–8.7 m depth in sand and shell bottom (Table 4). This depth interval of maximum bivalve abundance overlaps with Alheit’s (1981) study in Harrington Sound, which showed that 50−60% of bivalve mollusk biomass occurred from 6−12 m depth [32]. Similar to our study, Thomas (2003) and previous benthic sampling by the Bermuda Government revealed a zone from 3−9 m depth where calico clams were most abundant (Bermuda Department of Conservation Services, unpublished data). This latter zone coincides with the primary depths utilized by spotted eagle rays monitored in the 2007 and 2008 HS monitoring arrays, suggesting rays preferentially used depths that supported their primary food.

Our observation of spotted eagle ray predation on bivalves and gastropods in Bermuda is supported by previous studies that report this species as mollusk-specialized [6], [8], [9], [33]. As with other myliobatid rays, consumption of hard-shelled mollusks by the spotted eagle ray is mediated by its strong jaws and enlarged dental plates [6], [8], [9], [33], [34], [35]. Given the lack of shell extracted from the gut contents and gastric lavage, it was imperative that internal tissue photos of the available benthic fauna were obtained for diet analysis. The single individual that consumed gastropods (*Strombus costatus* and *Natica* sp.) was the largest ray by weight (73 kg) captured over the sampling period. The same individual also consumed calico clams and ark clams (*Anadara notabilis*), the latter of which was only observed in one other similarly large individual. Though the reduced sample size may not encompass the full dietary breadth of spotted eagle rays from Harrington Sound, the discovery of ark clams and conch in two larger individuals suggests that rays exhibit some degree of ontogenetic diet variation. This is supported by the work of Schluessel et al. (2011), who observed that spotted eagle rays from the Indopacific switch from a crustacean (i.e., malacostracans) diet to a hard-shelled mollusk diet with ontogeny. A comprehensive diet study encompassing all maturity levels is needed to elucidate these potential ontogenetic patterns in resource use.

Foraging patch selection in aquatic benthic vertebrates and invertebrates is commonly based on resource availability. In freshwater streams, foraging rates by longnose dace (*Rhinichthys cataractae*) on benthic cobble communities are significantly greater on stones with higher biomass of invertebrates, implying selection for these patches when resources are patchily distributed [36]. A variety of marine benthic fishes also appear to selectively forage in patches of high amphipod densities along intertidal mud flats of Nova Scotia [37]. Blue crabs (*Callinectes sapidus*) also exhibit high prey density-dependence when foraging on infaunal *Macoma balthica* with proportionally more clams consumed at higher density patches in enclosure experiments [38], [39]. While several studies suggest prey distribution influences habitat use in elasmobranchs, empirical evidence of this is sparse in the literature, likely due to the larger size of these predators and the difficulty in manipulating prey densities for experiments [40]. However, two related ray species that appear to exhibit prey density-dependent patch selection include the cownose and New Zealand eagle rays, which both respond non-linearly to increasing patch densities of bivalves when feeding in the natural environment [41], [42]. Large basking sharks, *Cetorhinus maximus*, also appear to select habitats based on threshold prey densities when foraging on plankton [43]. These threshold foraging behaviors observed in benthic and pelagic predators may be an adaptation to avoid net energetic losses in these highly mobile animals, as suggested by foraging theory. More studies that specifically address energetics in these predator-prey interactions are sorely needed.

Our combined findings from the enclosure and field manipulation experiments suggest that spotted eagle ray foraging may have little influence on clam gradients in Harrington Sound. Though rays appear to “cue” in on higher density areas of calico clam to forage, their feeding rates do not appear to be density-dependent. Spotted eagle rays held in an experimental enclosure responded to the ambient densities of their primary prey by removing significantly more clams at higher density patches. Moreover, measurable predation by rays was detectable in the field among manipulation patches situated among the highest density of clams in HS (Trunk Island), suggesting potential preference for these habitats. This apparent patch selection behavior exhibited by the spotted eagle rays conforms to optimality models that predict higher consumption of prey by predators in more “profitable” habitat types [44]. However, though consumption was significantly higher in high density patches, the enclosure experiment indicated that proportional mortality of clams was the same irrespective of density. The implication of this finding contrasts with Thrush et al. (1991), who postulated that foraging by *M. tenuicaudatus* may act to smooth distribution patterns of otherwise clustered patches of benthic invertebrates [45]. Our data suggest spotted eagle rays may not provide such a smoothing role.

Though spotted eagle rays demonstrated preferences for habitats that supported dense populations of important shellfish, given their combined lack of patch-depleting foraging behavior and relatively low population densities, it is unlikely that rays would completely deplete calico clam from Harrington Sound. The larger size, higher site fidelity and solitary foraging behavior of spotted eagle rays may account for their reduced consumptive effects on manipulated prey patches when compared to schooling cownose rays. Schools of fall-migrating cownose rays have been observed to deplete patches of bay scallop (*Argopecten irradians*) to complete extirpation off North Carolina [42]. Though spotted eagle rays are known to school during social interactions or in preparation for large-scale movement [12], in this study, all observations of foraging spotted eagle rays (field and enclosure) involved single individuals. Situated in subtropical-tropical environments, spotted eagle rays likely forage solitarily among more heterogeneous and stable benthic communities. Cownose rays, on the other hand, seasonally migrate in large schools along temperate inshore corridors where prey availability may be more episodic. To fully exploit these ephemeral resources, cownose rays may need to utilize stricter density-dependent foraging behaviors [46], which can lead to localized depletions and population sinks [42]. While we observed no such patch-depleting behaviors by spotted eagle rays, we caution that they are still important predators of protected inshore shellfish of Bermuda and preferentially forage in habitats supporting higher densities of calico clams. As such, rays could be detrimental to restoration programs that seed above ambient clam densities, which could potentially attract ray foraging. These effects can be exacerbated by reducing the density of ray predators in Bermuda such as large sharks.

Though evidence of habitat selection behavior in telemetered elasmobranchs has grown in the past few decades [47], [48], [49], [50] most studies have involved sharks and not rays. Moreover, few studies have been able to successfully link prey availability with the behavior of these free-ranging elasmobranchs [51], [52]. To our knowledge, this is the first study to simultaneously use telemetry and benthic mapping to examine predator-prey interactions in a batoid ray. Previous telemetry work on batoids have focused on the influence of abiotic factors on general movement patterns and habitat use [17], [18], [19], [20], [21], [53]. While batoid rays can be extremely mobile, their prey is generally constrained to benthic habitats, and thus they represent good candidate species for using telemetry to examine interactions with prey. With advances in mapping software (e.g. ArcGIS), a plethora of packages can be used in conjunction with telemetry research to estimate benthic habitat use in these animals and thus their potential impacts on benthic resources.

Our study demonstrates that the integration of individual-based study (i.e. acoustic tagging) with field sampling and experimentation can elucidate the habitat use and potential impact of large benthic mesopredators. Given the high mobility of mesopredators and relatively ephemeral nature in benthic systems, methods to derive residency patterns and fine-scale habitat use of these animals must be implemented in order to guide manipulative experiments capable of detecting foraging effects. In our example, we coupled active and passive telemetry with field surveys and enclosure/exclosure experiments to unveil the potential impacts of highly mobile fish on a relatively stationary mollusk species. While our approach is highly applicable to other predator-prey systems that include mobile predators and fixed prey, we caution that researchers should have a strong underpinning of the dynamics of their study system prior to implementing these experimental approaches.

## Supporting Information

Protocol S1 **Transmission scheme for pressure/motion sensor data from acoustic transmitters.**
(DOCX)

Protocol S2 **Methods for determining total available habitat at each receiver station.**
(DOCX)

Protocol S3 **Methods for determining long-term residency in Harrington Sound.**
(DOCX)

Protocol S4 **Towed hydrophone tracking survey methodology.**
(DOCX)

Protocol S5 **Technique for meeting assumptions of the Schnabel population estimation method.**
(DOCX)
