# Supplementary material for: Estimating the Potential Impacts of Large Mesopredators on Benthic Resources: Integrative Assessment of Spotted Eagle Ray Foraging Ecology in Bermuda
Source: PLoS One. 2012 Jul 3;7(7):e40227. doi: 10.1371/journal.pone.0040227 (PMC3388999; doi:10.1371/journal.pone.0040227)
Supplement: Protocol S1 — Transmission scheme for pressure/motion sensor data from acoustic transmitters. (DOCX) [file pone.0040227.s001.docx]

**Protocol S1.**

A motion transmission (value = 1) occurred when a transmitter was actively moving within the last minute of sensor logging, whereas an inactive transmission (0) was representative of an individual that had not moved within the last minute. The MA-PM-16_252 transmitters transmitted identification code and sensor information every 3 sec. Pressure sensor data was transmitted over four consecutive transmissions, and then interspersed with a motion sensor transmission (i.e., 20 pressure transmissions per min, 5 motion transmissions per min). These specifications equated to a battery life of 57 d for MA-PM-16_252 transmitters.
