# Supplementary material for: Estimating the Potential Impacts of Large Mesopredators on Benthic Resources: Integrative Assessment of Spotted Eagle Ray Foraging Ecology in Bermuda
Source: PLoS One. 2012 Jul 3;7(7):e40227. doi: 10.1371/journal.pone.0040227 (PMC3388999; doi:10.1371/journal.pone.0040227)
Supplement: Protocol S2 — Methods for determining total available habitat at each receiver station. (DOCX) [file pone.0040227.s002.docx]

**Protocol S2.**

Based on range tests, each hydrophone was assumed to have a 700 m detection radius. Hydrophone range and digitized bathymetry maps were used with the 3D Analyst extension in ArcMap 9.2 (ESRI, Inc.) to estimate the total amount of detectable water volume at each station within Harrington Sound. The detection volume was influenced by the presence of islands and/or shoreline habitat occurring in “line of sight” of 700 m buffered radii of each hydrophone. These acoustic shadowing regions were manually deducted from the total coverage at each hydrophone location in ArcMap to produce an adjusted detection volume estimate.
