# Supplementary material for: Estimating the Potential Impacts of Large Mesopredators on Benthic Resources: Integrative Assessment of Spotted Eagle Ray Foraging Ecology in Bermuda
Source: PLoS One. 2012 Jul 3;7(7):e40227. doi: 10.1371/journal.pone.0040227 (PMC3388999; doi:10.1371/journal.pone.0040227)
Supplement: Protocol S3 — Methods for determining long-term residency in Harrington Sound. (DOCX) [file pone.0040227.s003.docx]

**Protocol S3.**

Long-term residency in Harrington Sound (HS) was analyzed from the two gate-keeping hydrophones by subtracting time of arrival at H0 from the last detection at hydrophone H1 when detections were preceded and followed by detections from H1. Such a scenario indicated an interval where an animal had moved from Flatts Inlet (FI) to HS, and vice versa. This technique for estimating residency was confirmed through field tests where detection patterns of the gate-keeping hydrophones were examined after towing active transmitters behind a vessel from HS through FI and vice versa.
