# Supplementary material for: Estimating the Potential Impacts of Large Mesopredators on Benthic Resources: Integrative Assessment of Spotted Eagle Ray Foraging Ecology in Bermuda
Source: PLoS One. 2012 Jul 3;7(7):e40227. doi: 10.1371/journal.pone.0040227 (PMC3388999; doi:10.1371/journal.pone.0040227)
Supplement: Protocol S4 — Towed hydrophone tracking survey methodology. (DOCX) [file pone.0040227.s004.docx]

**Protocol S4.**

Each towed hydrophone tracking survey consisted of a single haphazard loop around Harrington Sound and into Flatts Inlet. The direction (clockwise vs. counterclockwise) of the tracking loop was randomized on the sampling day by flipping a coin. During tracking, we utilized two Lotek LHP_1 hydrophones mounted to the vessel with a PVC frame. The two hydrophones were attached to a Lotek Map600 receiver and dragged along the sides of the vessel at a speed of 4-7 km/hr. When animals were detected, the vessel was maneuvered into a circular pattern to create a synthetic array of hydrophones [[27](#_ENREF_27)]. Vessel positions were logged 1/sec with an onboard differential GPS (Ceeducer Pro, Bruttour International, Inc.) clock synched with the Map600 receiver.
