# Supplementary material for: Estimating the Potential Impacts of Large Mesopredators on Benthic Resources: Integrative Assessment of Spotted Eagle Ray Foraging Ecology in Bermuda
Source: PLoS One. 2012 Jul 3;7(7):e40227. doi: 10.1371/journal.pone.0040227 (PMC3388999; doi:10.1371/journal.pone.0040227)
Supplement: Protocol S5 — Technique for meeting assumptions of the Schnabel population estimation method. (DOCX) [file pone.0040227.s005.docx]

**Protocol S5.**

There are several assumptions associated with the Schnabel method for population estimation including: 1) the marked individuals (those taken during the first capture) have had time to mix into the population so that each marked individual is "equally catchable" as any unmarked individual, 2) marked and unmarked animals have an equal probability of being caught, 3) all marking must be done at the same time and the population size must remain the same between captures, and 4) the number of marked individuals does not change between the captures (no loss of marking, no mortality of marked individuals, no migration of marked individuals). Based on acoustic monitoring data, spotted eagle ray residence in Harrington Sound was variable. Thus, to address the criterion of a closed population in a species with known population flux, we adjusted our population size with the average Harrington Sound habitat use proportions from all individuals monitored with acoustic telemetry.
